# Eye Movement Measurement in Diagnostic Assessment of Disorders of Consciousness

**DOI:** 10.3389/fneur.2014.00137

**Published:** 2014-07-29

**Authors:** Windsor Kwan-Chun Ting, Jose Luis Perez Velazquez, Michael D. Cusimano

**Affiliations:** ^1^Injury Prevention Research Office, Keenan Research Centre for Biomedical Science, The Li Ka Shing Knowledge Institute, St. Michael’s Hospital, Toronto, ON, Canada; ^2^Institute of Medical Science, University of Toronto, Toronto, ON, Canada; ^3^Neurosciences and Mental Health Programme, The Hospital for Sick Children, Toronto, ON, Canada; ^4^Department of Paediatrics, University of Toronto, Toronto, ON, Canada; ^5^Division of Neurosurgery, Department of Surgery, St. Michael’s Hospital, Toronto, ON, Canada; ^6^Division of Neurosurgery, Department of Surgery, University of Toronto, Toronto, ON, Canada

**Keywords:** disorders of consciousness, eye movement, minimally conscious state, vegetative state, coma

## Abstract

We review the literature to appraise the evidence supporting or disputing the use of eye movement measurement in disorders of consciousness (DOC) with low levels of arousal or awareness, such as minimally conscious state (MCS), vegetative state (VS), and coma for diagnostic and prognostic purposes. We will focus on the effectiveness of each technique in the diagnostic classification of these patients and the gradual trend in research from manual to computerized tracking methods. New tools have become available at clinicians’ disposal to assess eye movements with high spatial and temporal fidelity. The close relationship between eye movement generation and organic dysfunction in the brain allows these tools to be applied to the assessment of severe DOC as a unique supplementary toolset. We posit that eye tracking can improve clinical diagnostic precision for DOC, a key component of assessment that often dictates the course of clinical care in DOC patients. We see the emergence of long-term eye-tracking studies with seamless integration of technology in the future to improve the performance of clinical assessment in DOC.

## Review

### Introduction

There are little means by which a clinician can assess and prognose clinical course in severe cases of disorders of consciousness (DOC), limiting effective care of patients. Furthermore, caregivers for DOC patients experience a higher level of anxiety, depression, and maladaptive psychological states compared with control participants and with themselves at the beginning of their caregiving period (1, 2). From both perspectives, it is important to have effective tools to make the best clinical decisions.

Disorders of consciousness are divided into several domains. Locked-in syndrome, minimally conscious state (MCS), vegetative state (VS), and coma are four major classifications (3). Each category of DOC is associated with its own clinical course and prognosis. Categorization of DOC has been repeatedly invaluable from a clinical perspective to make practical decisions about life support, palliative care, and/or treatment plan, but theory suggests that self-referential states such as consciousness are produced by coordinated diffuse network activations integrating multiple sets of different nuclei in the brain (4). If this is a reliable and accurate model, damage which significantly reduces consciousness will likely affect the brain areas necessary for successful eye movement. Quantifying this neurological damage may be useful and requires more than the simple qualitative appraisals of eye, sensory, and motor function.

The clinical gold standard for diagnosis of DOC is bedside behavioral assessment, in-hospital observation, and specialized robust scales such as the Coma Recovery Scale-Revised (CRS-R) (5), and neurocognitive assessment in advanced stages of recovery (6). Research has shown that neurophysiological monitoring may also be useful (7). The diagnosis from this assessment then dictates the clinical treatment plan and patient prognosis. However, it is difficult to make accurate decisions, partly due to the limited responsiveness and arousal of the patients, and partly due to the specialized training necessary to conduct a thorough neurological exam, and interpret neuroimaging and electrophysiology. A clinically standardized tool in the form of an eye movement measurement device, easily accessible and interpretable by all staff involved in the DOC patient’s care would help to supplement patient care. There are estimates of up to 40% misdiagnosis rate between VS and MCS (8). Traditionally, eye assessment in DOC has been limited to simple assessment of eye response. An acute care physician may look at opening or closing of the eyes in response to stimuli, pupil response to light, blink reflex, or the direction of spontaneous movement as part of clinical workup depending on the level of consciousness of the patient. Beyond qualitative appraisal, however, there are many more parameters of eye movements, such as their velocity, accuracy, and error rate, which can be easily measured with the right machines and may provide valuable diagnostic and prognostic information that is not currently considered by physicians during clinical workup.

Published in 1974, the Glasgow Coma Scale (GCS) qualitatively assesses eye, motor, and verbal responses with a maximum score of 15 indicating full arousal and awareness (9). Despite being the most commonly used assessment of conscious state today (10), outcome measures for the eye response component are limited to eye opening or closing in response to simple stimuli (9). The GCS is an example where advanced eye-tracking technology can provide much richer and detailed information, which could be used in conjunction with existing techniques for more refined diagnosis and accurate prognosis of severe DOC. The GCS score is also an easily accessible system for all personnel involved in the patient’s circle of care, and this has been reflected in the creation of clinical diagrams and visual flowcharts. Although no standardized equivalent exists for eye movements at the moment, as the technology is validated and more widely adopted, it is likely that similar resources would be created to lower the barrier to adoption for most health care personnel.

Further development of eye tracking and recording technology in the mid-1990s to sufficient temporal and spatial fidelity was needed before useful information could be obtained from recordings to make an informed assessment. Today, these eye trackers are commonplace in research of severe neurological disorders ranging from multiple sclerosis (11) to visuospatial neglect (12). The current research standard is the full desktop, which is limited in portability despite its power. Slowly, we are seeing the emergence of portable machines, which are worn on the head and able to measure eye movements on the sub-millisecond and sub-millimeter scale. Although this eye-tracking technology is used extensively in research, it is not used for clinical decision making.

The neuroanatomy responsible for many eye movements is well characterized in primates and humans (13–18). In humans, ocular motion is controlled by three sets of oculomotor muscles (15). Although they are the ultimate effectors of eye movement, computation for simple eye movements like saccades occurs over many levels of neural control connecting the cerebral cortices, through the midbrain structures, down to the lower brainstem where the desired eye movement is integrated and the final signal is issued (19).

Hence, an intricate connection exists between eye movement pathology and DOC. Generation of successful eye movements and a fully conscious state with high levels of arousal and awareness both depend on coordinated activity between multiple levels of neural control centers, including the cerebral cortex capable of modulating activity in most systems below it, the subcortical nuclei, midbrain and lower brainstem nuclei. Many studies have suggested that DOC may be reflected in eye movement pathology due to disruptions in these sensitive structural and functional connections.

Our knowledge of the neuroanatomy involved in DOC, although still young, has made great progress mainly through advances in functional neuroimaging of DOC patients (20, 21). Relatively novel techniques such as susceptibility/diffusion weighted imaging (S/DWI) and diffusion tensor imaging (DTI) have enhanced sensitivity to fine structural damage than canonical techniques (computerized tomography for example). Functional neuroimaging has quickly flourished and can assess transient fluctuations in regional cerebral blood flow and perfusion in the brain as well as functional integrity through neural connections, which may appear structurally intact otherwise. Advanced neuroimaging has been reviewed extensively as a tool to assess DOC and shows promise (20–25), but current clinical evaluation tools outside of neuroimaging remain limited in scope and power. Not all healthcare facilities have the financial resources and expertise to use neuroimaging for DOC assessment, but eye trackers are readily available, have lower cost of procurement, and are relatively more portable than MRI scanners. Eye movement measurement can therefore be a complementary evaluation tool for patients with severe DOC, or an alternative when the necessary neuroimaging resources are not available.

### Vestibulo-ocular reflex

The vestibular system and associated vestibulo-ocular reflex is well characterized (26). In DOC research, two different approaches have been used to elicit VOR. The traditional method is with caloric stimulation (27–33), in which warm or cold water is poured into the auditory canal unilaterally and quick phases of nystagmus are observed. Caloric VOR response has been associated with eventual recovery in several research and case studies (see Table 1 for details) (28–30, 33, 34).

**Table 1:** **Literature investigating eye movement measurement in disorders of consciousness**.

Citation	Sample size and DOC	Clinical utility of eye movements measurement	Relevant eye movement measured	Primary relevant outcome
**SECTION 1: PRIMARY RESEARCH STUDIES**				
Trojano et al. (35)	9 VS; 9 MCS; 11 HC	+	VT	On-target fixation proportion is different between MCS and VS
Weiss et al. (33)	26 VS	+	Caloric horizontal VOR, NY	Fast component of NY during VOR present in all patients who recovered consciousness; only one present in patients who remained unconscious
Candelieri et al. (36)	9 VS; 13 MCS	+	VP	VP elicited in 62% MCS but only 33% VS; VP activity fluctuates over a day
Dolce et al. (37)	395 VS	+	VT	VT in 73% of VS patients; appearance of VT indicative of higher GOS outcome
Bruno et al. (38)	10 VS; 39 HC	[N]	VF	VS patients exhibited altered PET metabolic dysfunction compared to HC; VF +ve metabolism did not differ from VF −ve metabolism
Balazs et al. (39)	14 PVS	+	SBEM	PVS plus recovery patients show stronger pre-slow ballistic eye movement gamma minimum than PVS minus recovery patients
Schlosser et al. (34)	5 CO (All GCS 3)	+	Galvanic VOR	Patient with no response to galvanic stimulation later brain dead; patients with spontaneous and VOR response to galvanic stimulus later recovered
Oksenberg et al. (40)	11 VS; 6 HC	−	Sleep REM phasic activity	REM phasic activity in VS unrelated to recovery
van den Berge et al. (31)	30 CO	[N]	Inter-rater reliability of VOR-caloric	Kappa within range of 0.46–0.49, indicating moderate agreement
Mueller-Jensen et al. (30)	81 CO	+	Oculocephalic response, VOR	Absence of VOR is indicative of −ve outcome; presence of VOR is indicative of +ve outcome, 67% of the time
Born et al. (29)	109 sustained LOC (GCS ≤7)	+	Oculocephalic response, VOR	Via logistic discriminant analysis of outcome, integration of VOR into assessment improves outcome prediction at 6 months
Yagi and Baba (27)	86 CO	−	Caloric VOR	Auditory–Brainstem Response (non-eye) was more effective than caloric VOR in prognosis of CO patients. Furthermore, not all patients who lacked VOR response had poor outcome
Braakman et al. (41)	305 CO	+	Reflex and spontaneous reflex eye movement	Reflexive and spontaneous eye movement is predictive, along with age in decades, duration of coma, GCS score, and pupillary response of GOS outcome in CO patients 6 months later
**SECTION 2: CASE STUDIES/SERIES AND LETTERS**				
Trojano et al. (42)	13 VS/UWS, 13 MCS; 13 HC	+	VT	MCS patient ratio of on-target:off-target fixation for pictures of participants’ relatives higher than circle or parrot stimulus. Proportion of on-target fixation in VS below chance; in MCS above chance
Schnakers et al. (43)	60 post-CO; of these, 29 VS, 7 MCS	+	CRS-R	Scaled assessment of visual tracking in CRS-R more sensitive than FOUR score, identified seven more patients who were in MCS
Kane et al. (44)	60 CO	+	Ocular micro-tremor	Oculogram Grade (OG) (based on graded assessment of conjunctive micro-tremor) positively correlated with GCS score at time of assessment and GOS outcome at 3 months
Leigh et al. (28)	6 CO, 4 PVS, 6 HC	+	Oculocephalic response brought by VOR	Post-VOR midline drift time constant ≥10 s in HC; ≤1.5 s in sLoC; ≤0.5 in PVS
van Woerkom et al. (45)	1 CO; 60 TBI patients later in DOC	+	Caloric nystagmus	Abnormal saccadic oscillations in 10 TBI patients associated with poor GOS 1/6 months post-injury

*MCS, minimally conscious state; VS, vegetative state; PVS, persistent vegetative state; UWS, unresponsive wakefulness syndrome; CO, comatose state; sLoC, sustained loss of consciousness; pt/pts, patient(s); HC, healthy control; SCC, saccade; VOR, vestibulo-ocular reflex; VP, visual pursuit; VF, visual fixation; VT, visual tracking; SBEM, Slow Ballistic Eye Movement; REM, Rapid Eye Movement; NY, Nystagmus; [N], Neutral; TBI, traumatic brain injury; GCS, Glasgow Coma Scale; GOS, Glasgow Outcome Score; FOUR, Full Outline of UnResponsiveness Scale; CRS-R, Coma Recovery Scale-Revised*.

The second method involves electrical (galvanic) stimulation, in which the signal can be modulated based on the research question or clinical assessment goal, thus having a greater versatility than the traditional caloric stimulation method (34). This has been used by one group to assess patients in VS and MCS (34). They found that quantitative VOR response from galvanic stimulation recorded using a computer was able to stratify clinical outcome for a group of five comatose patients all with GCS of 3 on admission (34). Although only limited conclusions can be drawn from five cases, it illustrates the potential for computer-based eye tracking for measuring VOR in patients who have a low responsiveness and arousal. More studies are needed to demonstrate how specific these effects are between MCS and VS patients. Not all research positively suggested that VOR is effective in DOC assessment, however. Yagi and Baba (27) investigated 86 comatose patients using caloric VOR and auditory–brainstem responses (ABR) (an electrode-based measurement). They found that the ABR was more effective in predicting outcome in coma, but not all patients who were unable to exhibit VOR had a poor outcome (27). All considered, VOR could be a robust tool for assessment and prognosis in DOC, and is currently the eye measurement of choice compared to other movement types.

### Visual fixation and pursuit

Visual fixation and pursuit are the second most feasible measure of eye movement in DOC. Successful visual fixation is indicative of functional recovery (43). Furthermore, previous research have measured visual pursuit and tracking with high throughput eye trackers and there is evidence suggesting that VT performance can effectively differentiate between MCS and VS states. For example, Trojano et al. (35) conducted an experiment where moving shape stimuli were connected to a computerized eye tracker to assess the characteristics of visual tracking in 9 VS, 9 MCS, and 11 healthy control participants. They found that the proportion of off-target to on-target fixations were significantly different between the VS and MCS patients. Furthermore, there was a clear qualitative difference in the path of visual tracking between the three groups that could easily be identified by a clinician or nurse. Although simple assessment of visual fixation does not require eye-tracking technology, this is an example of how the technology can help make assessments more precise, accurate, and effective.

Candelieri et al. (36) studied visual pursuit in 9 VS and 13 MCS patients using the scaled CRS-R, and found that in 63% of MCS patients effective visual pursuit could be elicited, whereas only 33% of VS patients were able to initiate them. They were also one of the first to report that visual pursuit performance fluctuates over the day in DOC patients. Finally, a study by Bruno et al. (38) coupled advanced neuroimaging with scaled visual tracking as measured by the CRS-R. They found that patients in the VS had altered metabolic function in PET neuroimaging compared with the healthy control group. Studies discussed here, which quantitatively measured visual tracking behavior, used simple measures such as the proportion of successful visual tracking time on-target or off-target. However, many other variables can be obtained from a computerized trace, and future studies could delve more deeply to identify more sensitive and specific biomarkers of conscious state in these patients with the data obtained from the eye-tracking technology.

### Saccades and rapid eye movements

Rapid eye movements (REMs) such as saccades allow us to quickly focus on objects in our environment. The saccadic generation system is well characterized in humans (13, 14, 16). Signal generation and final integration of the oculomotor command takes place in the brainstem, but upstream modulatory centers in the midbrain (superior colliculi) and cerebral cortices (frontal and parietal eye fields) play critical roles in the accurate generation of different types of saccades (19, 46). Saccades can be generated from memory, in response to novel visual stimuli, or directed away from a certain novel visual stimulus (19). The saccadic system is well integrated between different levels of neural control. Furthermore, highly portable saccadometers have been designed that can be worn on the patient’s head, with the visual stimulus projected onto a white wall (47).

Saccades would be the ideal system of eye movement to measure, since they are readily quantifiable and have mapped neuroanatomical substrates. However, reliable measurement of saccades in response to memory or novel visual stimuli is difficult in DOC patients because many experimental paradigms require conscious awareness. There has been little research looking specifically at saccadic measures in DOC, except for quick saccadic oscillations (39). Microsaccades (small involuntary jerks of the eye) may be useful as an alternative for eye movement assessment of visual attention in the future (48–50), but their role in DOC, if any, have yet to be established.

### Other eye movements – quick phases of nystagmus, ocular micro-tremors, REM

Nystagmus is an involuntary eye movement associated with saccades requiring little cortical integration, which has been used in the assessment of DOC patients after VOR stimulation (45). Ocular micro-tremors (discussed in more detail below) have also been investigated in DOC patients with analog machinery, but not with computerized eye-tracking software (44). Very few research studies have measured REM during sleep in VS patients. Oksenberg et al. (40) conducted a study with 11 VS and 6 healthy control participants, but did not find a significant relationship with recovery (40).

### Validation of eye movement measurement in DOC

Only one study was found to assess reliability of eye movement measurement in DOC, and no studies assessing validity were identified. van den Berge et al. (31) assessed the inter-rater reliability of vestibulo-ocular reflex monitoring in 30 comatose patients (31). This study used a scaled measure of VOR from one (no reaction) to four (full nystagmus). The VOR responses were recorded on film and analyzed after the experiment by experienced neurologists. The kappa (inter-rater reliability) ratings for spontaneous eye movements and oculocephalic responses were 0.46 and 0.49, respectively indicating moderate agreement (31). More studies investigating the sensitivity, specificity, reliability, and validity of eye movements measurement in DOC patients are required if these methods are to gain widespread acceptance in medical practice.

### Case studies, case series, and letters

Overall, there have been few clinical case studies and letters investigating eye movement measurement in DOC. Trojano et al. (42) conducted a study in which 13 VS, 13 MCS, and 13 healthy control participants viewed circles, parrot pictures, and pictures of their relatives as stimuli in an eye-tracking paradigm for visual pursuit and tracking (42). They found that in MCS patients, the ratio of on-target to off-target fixations for pictures of their relatives was higher than for the other two stimuli, indicating there is some visual processing and affective salience associated with the picture that is not present in VS patients (42). A natural progression from this study would be to investigate multi-sensory stimuli, which may combine auditory and visual domains, for example, for a more nuanced approach and enhanced sensitivity to detect differences between MCS and VS patients.

Schnackers et al. conducted a study in 2006 in which 60 post-comatose patients (of these, 29 VS and 7 MCS) were graded according to the CRS-R. They found that scaled assessment of visual tracking was more sensitive in the CRS-R to outcome than the Full Outline of UnResponsiveness (FOUR) score, which has less detailed eye response assessment (43). Here, we see the advantage of scaled assessment over simple appraisal of eye movement; a similar advantage can be gained when considering electronic eye movement measurement over scaled responses.

Kane et al. (44) reported a case series of 60 comatose patients whose ocular micro-tremor was assessed using an electrooculogram. By creating an “oculogram score” from the recorded eye tracings based on the level of conjunction of eye movement, they correlated ocular micro-tremor activity with GCS score at time of recording and GOS score at 3 months, with positive results (44). Leigh et al. (28) found that temporal characteristics of the post-VOR midline drift back to center could differentiate healthy controls, patients in persistent VS, and coma (28). As early as 1984, van Woerkom et al. reported that abnormal saccadic oscillations associated with caloric stimulation in TBI patients was associated with poor GOS outcome 1 and 6 months post-injury (45). Most of these examples have shown that quantitative measurement of eye movements has clinical utility either in diagnosing DOC or in assessing long-term prognosis of these patients. Table 1 summarizes our findings.

## Trends in Eye-Tracking Technology

All types of eye movement responses are easily measured using a modernized computerized eye tracker, including VOR, visual fixation, smooth pursuit, nystagmus, and saccades. Commercial companies have developed advanced eye-tracking systems for use in research, such as the EyeLink^®^ 1000 by SR Research (51) or the IVIEW XTM High Speed Eye Tracker by SensoMotoric Instruments (52). These high speed advanced eye trackers are able to measure many parameters at once with sub-millisecond temporal resolution and spatial precision to within a 10th of a degree, all with sampling frequencies in the thousands of hertz (51). Recent developments such as the IRIS Infrared Limbus Eye Tracker (53) by Skalar and the Portable Saccadometer by Ober Consulting (47) have introduced head-mounted eye tracking, which increases portability of the instrument and feasibility for clinical adoption. Many of the advanced systems are programable and the portable units allow measurement parameters (i.e., stimulus latency, location, frequency) to be tailored for clinical applications.

With technological progress, we have been able to measure spontaneous eye movements or ocular responses to stimuli with greater accuracy and precision. Since the 1990s, analog eye-tracking technology such as the electrooculograph has gradually been replaced by digital methods connected to high throughput computers. Furthermore, a clear trend has emerged toward miniaturization and enhanced temporal/spatial fidelity. Over time, these trends are expected to continue, such that advanced eye tracking will be easier to integrate into clinical practice by eliminating physical barriers such as size and portability while maintaining high quality for research or clinical applications. We expect these trends to continue with advances in wearable consumer technology (e.g., Google Glass analogs, fitness trackers, smartwatches).

## Research Perspectives and Future Directions

Currently, there are a limited number of studies supporting these measurements in clinic environment, and only one validation study has been found in MEDLINE. Despite this, the available evidence is compelling. The results of the literature review suggest that majority of the eye movement recording conducted in research currently are limited to VOR or other simple responses, which are quick to conduct, quantify, and interpret. In the future, however, as battery life associated with portable technology is extended, we should see the emergence of long-term eye-tracking studies, which may detect patterns over extended periods of time (over the course of several days, weeks, or months) unfeasible with current devices. This will open new domains in research and clinical decision making for these patients, since patterns may emerge from protracted, natural eye-tracking recording in patients, which is not detectable in short timepoints.

## Practical Perspectives

Currently used approaches such as the GCS or the FOUR score are effective and are simple and quick enough to perform while still being sufficient to make diagnostic decisions. The eye trackers that are clinically useful today, much like the useful computers of several decades ago, are large and unwieldy, requiring the patient to be in a specific body position (e.g., sitting upright) and are not suited for use in patients with secondary neck injuries due to head trauma (limited neck movement). With more robust research, results supporting the use of eye tracking in assessing DOC in the future, in tandem with the miniaturization trends discussed earlier, we see that the practical benefits of improved outcome assessment will outweigh the costs associated with adopting new technology and framework for assessment. Neuroimaging went through a similar process from clinical acceptance to adoption since its development several decades ago, but it is now part of routine clinical assessment and care. A similar level of research and development needs to take place for advanced eye measurement in DOC patients moving forward. Currently, another important barrier to clinical adoption include limited validation studies demonstrating the diagnostic power of eye movement measurement in DOC, as discussed earlier. These developments and studies will likely need to take place before we can expect to see widespread clinical adoption of eye movement assessment.

The clinical and research ethics councils in health organizations need to discuss issues of patient consent and develop a framework for deployment of these tools, but integration of eye-tracking measurement tools should not completely replace what is currently available. There is critical value in the clinical assessment and the physician’s appraisal of conscious state. Although eye movement measurements may prove to be an effective diagnostic tool, training critical care physicians and nurses for more thorough neurological examinations and clinical expertise is still very important and is likely to have the largest immediate impact on reducing misdiagnosis rate. A clinician’s judgment obtained from time-tested tools should not be replaced, but further augmented by this technology. In the final analysis, integration and adoption of eye-tracking tools and methods should not be a barrier, but rather a helpful aid toward better care and reduction of misdiagnosis for any patient in a DOC.

## Limitations

There are limitations to our literature search and interpretation. We only reviewed results from Ovid MEDLINE. Study quality was not systematically assessed. Diagnostic randomized controlled trials were not found (54), which might identify whether eye-tracking assessment provides any advantage over the contemporary gold standard of behavioral assessment. Finally, there were very few studies which quantitatively assessed the prognostic ability of eye tracking in DOC (see Table 1). Although some studies performed correlational analyses between measurement and GOS outcome, more studies along this avenue are needed to see if eye tracking has any prognostic relevance or if it is only useful as a diagnostic classifier.

## Conclusion

### An integrated perspective

Richer information about how eye movement/response is affected will be instrumental in advancing clinical assessment of DOC in the future. As medical technology becomes quicker, more accurate, and miniaturized, the integration of advanced measurement tools like the ones discussed into our daily lives will quickly become seamless. It is imperative, therefore, that a clinical framework be set in place early so that the implementation of these tools in medicine is not a barrier unto itself. Creating a strategic plan integrating economic, research, logistic, and ethical aspects is essential for successful transition from simple appraisals of eye response, through scaled assessment of eye movement, to widespread adoption of quantitative eye measurement by the bedside.

### A vision for the future

We can envision a future in which technology to measure eye movements is embedded into the process of medicine so well that it becomes invisible – physicians will have crucial human interactions with patients, but their judgment will be aided by semi-autonomous measurement machines, which track eye movements over their entire stay in the hospital, and generate a report with recommended diagnostics and projected clinical outcome. Clinical decision making can be augmented through a standardized and validated scale based on simplifying heuristics obtained from the eye-tracking device. All of this tracking will be conducted with minimal physician input. The eye-tracking machine itself could take the form of a wireless contact lens that the patient wears, offering the optimum combination of portability, power, sensitivity, and non-invasiveness. This vision may not be too far off – innovators in silicon valley have already developed eye-tracking add-ons for Google Glass, the *de facto* vanguard in wearable computing today (55).

In conclusion, research evidence supports integration of quantitative eye movement measurement in improving diagnostic precision in of DOC. However, more validation studies are required to assess prognostic relevance of eye tracking. It will also require a coordinated effort between disciplines between frameworks of medicine and research to bring this beneficial assessment methodology to clinical care of DOC.

## Author Contributions

Windsor Kwan-Chun Ting: “substantial contributions to the conception or design of the work; drafting the work; final approval of the submitted version; agrees to be accountable for all aspect of the work and ensures that any questions related to the accuracy or integrity of any part of the work are appropriately investigated and resolved.” Dr. Jose Luis Perez Velazquez: “substantial contribution to the conception of the work; critically revising the work for important intellectual content; final approval of the submitted version; agrees to be accountable for all aspect of the work and ensures that any questions related to the accuracy or integrity of any part of the work are appropriately investigated and resolved.” Dr. Michael D. Cusimano: “substantial contribution to the conception of the work; critically revising the work for important intellectual content; final approval of the submitted version; agrees to be accountable for all aspect of the work and ensures that any questions related to the accuracy or integrity of any part of the work are appropriately investigated and resolved.”

## Conflict of Interest Statement

Windsor Kwan-Chun Ting is conducting graduate thesis work using eye-tracking technology in assessment and prognosis of traumatic brain injury. Dr. Michael D. Cusimano has received Canadian Institutes of Health Research Funding to use eye movements to assess traumatic brain injury. Jose Luis Perez Velazquez declares that the research was conducted in the absence of any commercial or financial relationships that could be construed as a potential conflict of interest.

## Appendix

Ovid MEDLINE was searched for peer-reviewed literature meeting specific inclusion/exclusion criteria.

INCLUSION
Primary clinical studies/case series/letters.
Study conducted in humans.
Patient group must have sustained pathological sustained loss of consciousness (VS, PVS, MCS, or Coma).
Measurement must be quantitative or scaled and conducted during DOC.
Abstract or full text written in English.

EXCLUSION
Duplicate results
Loss of consciousness due to intoxication (intoxication itself may adversely affect eye movements).
Non-pathological loss of consciousness (e.g., sleep, g-forced induced, anesthesia, Valsalva maneuver loss of consciousness studies).
Studies of locked-in syndrome (patients present with high arousal and awareness).
Study published prior to 1980.
Reviews or Opinion Articles.

MEDLINE Search Algorithm
Ovid Medline was queried on 20 November 2013 as follows.
Database: Ovid MEDLINE(R) (1946 to November Week 1 2013), Ovid MEDLINE(R) In-Process and Other Non-Indexed Citations (November 19, 2013).

**Search strategy:**
exp consciousness disorders/or exp consciousness/(44522)
exp eye movements/(39713)
1 and 2 (313)
remove duplicates from 3 (308)
limit 4 to (English language and humans and year = “1980 − Current”) (130)
limit 5 to “review” (20)
5 not 6 (110).

Each abstract was screened with full-text if available. Studies which did not fulfill the inclusion criteria, met the exclusion criteria, or studies for which the full text was not available and the abstract alone was insufficient were removed (*N* = 91). Five articles were automatically removed as duplicates during the search. Thirteen primary research studies were included in the final review. Five case studies and letters were also included, but grouped separately in Table 1.
